# Flood exposure and poverty in 188 countries

**DOI:** 10.1038/s41467-022-30727-4

**Published:** 2022-06-28

**Authors:** Jun Rentschler, Melda Salhab, Bramka Arga Jafino

**Affiliations:** 1 The World Bank, Washington, DC USA; 2 Payne Institute for Public Policy, Denver, CO USA; 3 Center for Advanced Spatial Analysis, University College London, London, UK; 4 Deltares, Delft, The Netherlands; 5 Faculty of Technology, Policy, and Management, Delft University of Technology, Delft, The Netherlands

**Keywords:** Natural hazards, Developing world

## Abstract

Flooding is among the most prevalent natural hazards, with particularly disastrous impacts in low-income countries. This study presents global estimates of the number of people exposed to high flood risks in interaction with poverty. It finds that 1.81 billion people (23% of world population) are directly exposed to 1-in-100-year floods. Of these, 1.24 billion are located in South and East Asia, where China (395 million) and India (390 million) account for over one-third of global exposure. Low- and middle-income countries are home to 89% of the world’s flood-exposed people. Of the 170 million facing high flood risk and extreme poverty (living on under $1.90 per day), 44% are in Sub-Saharan Africa. Over 780 million of those living on under $5.50 per day face high flood risk. Using state-of-the-art poverty and flood data, our findings highlight the scale and priority regions for flood mitigation measures to support resilient development.

## Abstract (web summary)

Floods are most devastating for those who can least afford to be hit. Globally, 1.8 billion people face high flood risks; 89% of them live in developing countries; 170 million of them live in extreme poverty making them most vulnerable.

## Introduction

Globally, natural shocks are estimated to cause an average of over $300 billion in direct asset losses every year; this estimate increases to $520 billion when considering well-being (or consumption) losses^1^. While each country faces its individual set of natural hazards, including cyclones, earthquakes, or wildfires, floods are among the leading threats to people’s livelihoods and affect development prospects worldwide^2^. Especially in lower-income countries—where infrastructure systems, including drainage and flood protection, tend to be less developed—floods often cause unmitigated damage and suffering^3^. Recent disastrous floods in countries as diverse as Nigeria, Bangladesh, Vietnam, the United States, and the United Kingdom illustrate that the threat is a global reality. Rare, major floods and smaller, frequent events alike can revert years of progress in development^4^ and poverty reduction. Understanding the scale and distribution of risks is crucial for devising targeted mitigation measures and allocating adequate resources.

While the threat is already substantial, several ongoing trends could result in significant increases in flood risks in coming years. For a high-concentration climate change scenario, estimates from 11 climate models converge to the conclusion that flood frequencies in Southeast Asia, East and Central Africa, and large parts of Latin America could increase substantially by 2100^5^. Even in an optimistic climate change scenario (RCP 2.6), sea levels are estimated to rise up to 0.55 m by 2100, putting especially large coastal cities at risk^6^. Land subsidence, often caused by unsustainable ground water extraction and drainage, has been shown to increase coastal flood risks at a rate four times faster than sea level rise^7^.

Flood risks are also driven by socioeconomic change, as the number of people, assets, and value of economic activities increase over time^3^. By one estimate, in the absence of risk-mitigating measures, socioeconomic growth could result in the absolute damages from flooding to increase by a factor 20 by 2100. Considering the compounded effect of these drivers in the world’s 136 largest coastal cities, one study has shown that population and asset growth, climate change, and subsidence are likely to contribute to a drastic increase in global average flood losses, from $6 billion per year in 2005 to over $60 billion in 2050^8^.

Recognizing the severe impacts of disasters on socioeconomic development, many flood exposure assessments have been conducted at local and national scales, often leveraging the recent availability of high-resolution flood, asset, and population maps, enabling increasingly accurate risk assessments. Yet, local studies have focused predominately on high-income countries like the European Union, United States, and Japan, not least due to data availability and the large economic values at risk^9,10^. While studies exist for developing countries, attention is focused on large economic centers like Jakarta, Dhaka, Dar es Salaam, Accra, and Ho Chi Minh City^11–16^; few systematic assessments exist for the least developed countries and subregions, where floods are likely to have the most devastating impacts on livelihoods.

Overall, there is limited evidence on the global scale of flood exposure and how it relates to the incidence of poverty. Previous global flood risk assessments suffer from multiple limitations. By using global historical inventories of recorded flood events (e.g., from EM-DAT), studies have estimated exposure indicators at the country level^17^. Yet, the lack of data on the spatial distribution and coincidence of flood risk and populations means that this approach does not allow a robust estimation of exposure headcounts^17,18^﻿. A more recent study documents the worrying trend of increasing flood exposure using satellite data for 2000 to 2018, though omits at-risk populations who remained unaffected during the study period and many events that remain undetected by the satellite observations^19^.

Studies that use relatively coarse (by current standards) spatial resolution flood hazard data tend to only represent major fluvial floodplains. This means they are unable to capture pluvial flood risk and flooding along secondary rivers, and thus drastically underestimate exposure^3,5,20,21^. One study projects that the global number of flood-exposed people will reach 1.3 billion by 2050^20^, but our study shows that this threshold has already been exceeded by at least 39%. This illustrates the importance of high-resolution data to capture the highly localized nature of flood risks, and the tendency of people to avoid settling in the riskiest locations^22^. Other global studies have only focused on certain types of flood, rather than assessing the combined risks from fluvial floods (rivers exceeding their capacity due to excessive precipitation), pluvial floods (surface water build-up due to extended precipitation and insufficient drainage), and coastal floods (due to tidal or storm surges, or sea level rise)^2,23–27^. For instance, a recent study conducted a detailed global assessment of the risk of sea level rise to the world’s coastal population^28^, estimating that over 190 million people live in areas that could be inundated by sea level rise by 2100; but it does not consider inland flood risks. Other studies have only assessed risks for a subset of countries, falling short of full global coverage^22^. Most importantly, none of the existing global studies consider the intersection between flood exposure and poverty incidence, which is a crucial indicator for people’s vulnerability, resilience, and ability to cope with and recover from floods^1^. This study addresses these gaps.

We find that about 1.81 billion people, or 23% of the world population, are directly exposed to inundation depths of over 0.15 meters. This would pose significant risks to lives and livelihoods, especially of vulnerable population groups. The majority (1.24 billion) are located in South and East Asia, where China (395 million) and India (390 million) account for over one-third of global exposure. Low- and middle-income countries are home to 89% of the world’s flood-exposed people. Of the 170 million who face high flood risk and extreme poverty (living under $1.90 per day), 44% are in Sub-Saharan Africa. At least 780 million people face high flood risk, while living on less than $5.5 per day. We conclude that the number of people living in poverty and under severe flood risk is substantially higher than previously thought. Moreover, they are concentrated in vulnerable regions that face compounding risks from climate change, sociopolitical instability, and resource constraints that hamper effective risk management. By offering global, yet disaggregated, insights on flood risk exposure and poverty incidence, this study highlights the scale of the needs and priority regions for flood risk mitigation measures that can safeguard livelihoods and prevent prolonged adverse impacts on development.

## Results

**Fig. 1:**
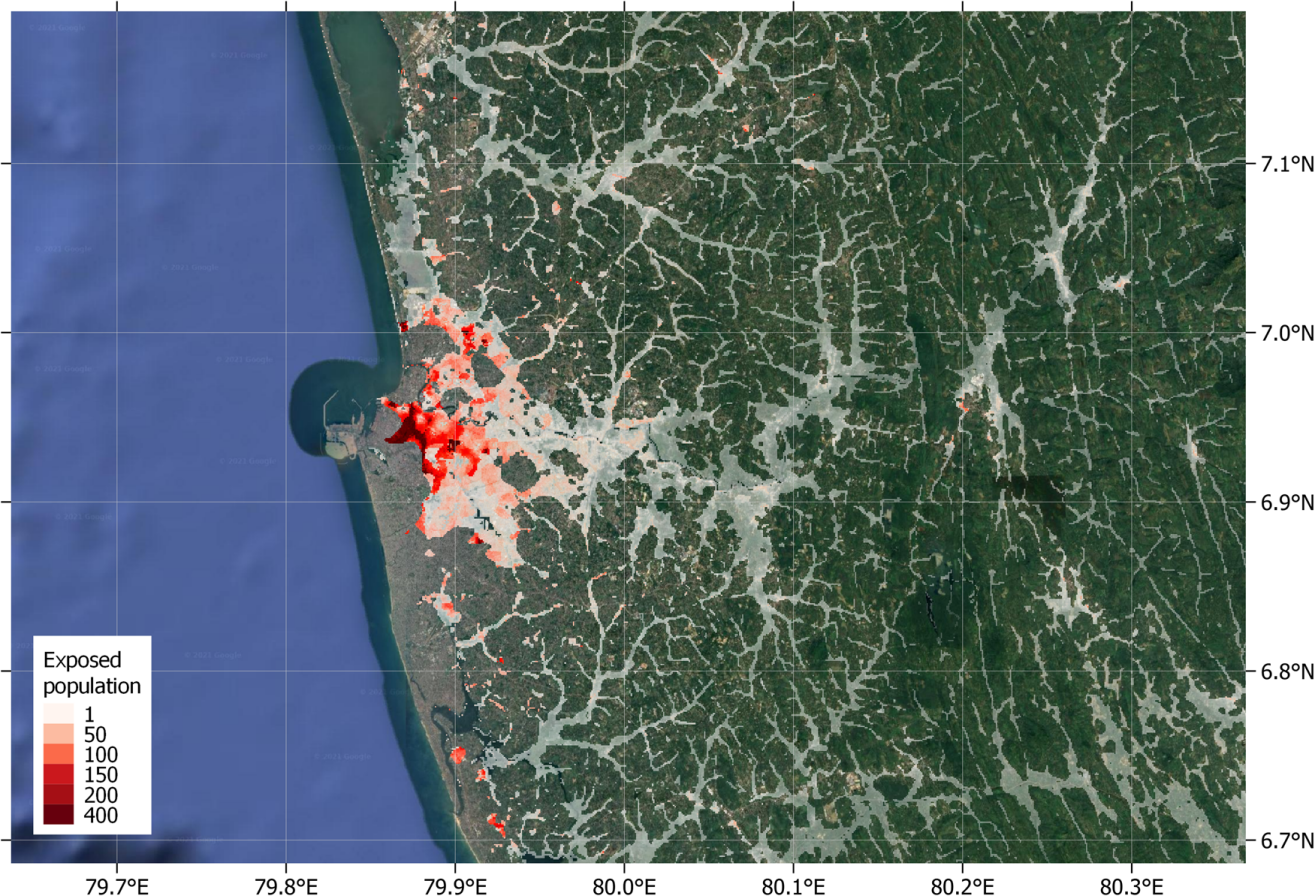
Flood exposed populations in the metropolitan region of Colombo, Sri Lanka. Highlighted areas correspond to populated locations with significant flood risk as identified in this study. White highlights correspond to low population density, while red highlights show densely populated areas. Legend numbers denote number of flood exposed people per 3 arcsecond pixel. (Image: Google, ©2022 TerraMetrics).

Here we present results from a high-resolution global exposure assessment for 188 countries, reaching within rounding errors of the entire world population. We assess people’s exposure to all current flood risks—that is, pluvial, fluvial, and coastal flooding. Flood data from Fathom-Global 2.0 are based on latest generation terrain and hydrographic models, while population density uses WorldPop 2020 maps calibrated on census and satellite data (Fig. 1). The global coverage of these datasets enables an overlay analysis with 3 arcseconds resolution (equivalent to about 90 × 90 meters at the equator), providing a more granular assessment than previous studies and eliminating the need for analytical assumptions besides the ones employed for producing the datasets. In addition, we use the latest edition of the World Bank’s Global Subnational Atlas of Poverty (GSAP), which harmonizes household survey data and offers poverty estimates with global coverage and statistical representativeness at the subnational level. Full technical details on data and computational process are provided in the “Methods” section.

### Global and regional flood exposure

Our estimates show that globally, 1.81 billion people (23% of the world population) live in locations that are exposed to a significant level of flood risk, facing inundation depths greater than 0.15 meters in the event of a 1-in-100-year flood, or at least medium risk (Fig. 1). In other words, considering a global population of 7.9 billion^29^, almost one in four of the world’s people are exposed to significant flood risk.

**Fig. 2:**
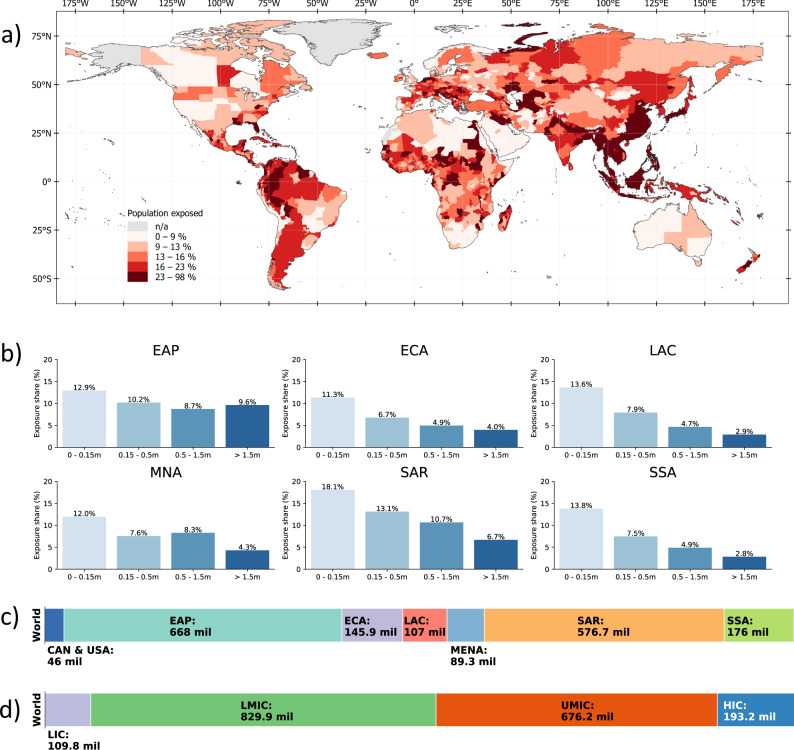
Population exposed to floods. **a** shows the percentage of population exposed to at least medium-level flood risk at the subnational level. **b** displays the percentage of population exposed to different levels of flood risk in each region. **c**, **d** show the total number of people exposed to at least medium-level flood risk based on geographical region and countries’ income classification, respectively. EAP East Asia and Pacific, ECA Europe and Central Asia (ECA), SAR South Asia region, SSA Sub-Saharan Africa, MNA Middle East and North Africa, LAC Latin America and the Caribbean, CAN & USA United States and Canada, HIC high-income countries, UMIC upper middle-income countries, LMIC lower-middle-income countries, LIC low-income countries.

Regionally disaggregating global exposure headcounts, it becomes apparent that flood risks are particularly prevalent in certain regions. At 668 million people, the East Asia and Pacific region has the highest number of people exposed to significant flood risk, corresponding to about 28% of its total population. In the South Asia region, 576 million people are exposed to significant flood risk (about 30.4% of the population). Between 9–20% of the regional populations of Sub-Saharan Africa, Europe and Central Asia, Middle East and North Africa, Latin America and the Caribbean, and the United States and Canada are exposed to high flood risk. Figure 2 provides a full breakdown of regional exposure estimates in absolute and relative terms. In East Asia and Pacific, South Asia, and the Middle East and North Africa, regional exposure is driven by single countries, namely China, India, and Egypt.

Our results also show that 1.61 billion (89%) of the world’s flood-exposed people live in low- and middle-income countries and about 193 million (11%) live in high-income countries (Fig. 2d). Considering that flood-exposed populations in high-income countries are more likely to benefit from flood protection systems, social postdisaster assistance, and other risk management support, these figures highlight the significant risks faced by developing countries. Full country-level results are provided in Supplementary Table 1.

### Countries with the largest flood-exposed populations

**Fig. 3:**
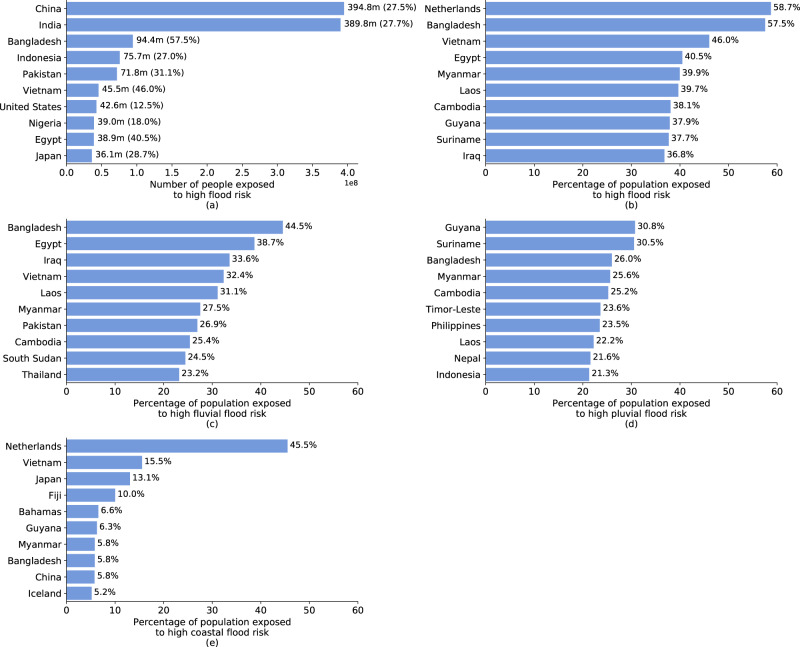
Absolute and relative population exposure at country level. **a** shows the ten countries with highest absolute number of people exposed (and as a percentage of the total population in parentheses). **b** shows the ten countries with highest relative population exposure. **c**–**e** show the ten countries with highest relative population exposure to different kinds of flood risks. Note: Countries or territories with populations of under 100,000 are omitted from this figure, in particular Andorra (24.3%, **d**) and Cayman Islands (6%, **e**).

Several countries stand out with particularly large populations directly exposed to high flood risk (Fig. 3a); and several factors explain this picture. Evidently, more populous countries are more likely to have large numbers of people living in direct exposure to flood risk. The two most populous countries, India and China, have the highest absolute exposure headcounts with 390 million and 395 million, respectively, and account for about one-third of all people exposed to flood risk globally. Yet, geographical features and urbanization patterns can drastically increase the size of exposed populations. The top 10 countries in terms of absolute exposure headcounts feature countries in which large population groups are concentrated along major river systems (e.g., Bangladesh, Egypt, Vietnam) or in coastal regions (e.g., Indonesia, Japan).

However, focusing on absolute exposure headcounts risks overlooking countries with smaller populations yet large relative exposure. Figure 3b presents the top 10 countries in terms of percentage of population exposed to high flood risk, in all of which over one-third of the population is flood-exposed. The Netherlands has the world’s highest relative exposure to flood risk, with 58.7% of the population living in areas that would face inundation depths of over 15 cm in the event of a 1-in-100-year flood without considering flood protection systems. The country has some of the world’s most comprehensive flood protection systems, with protection against extreme events of up to 1-in-10,000-year return periods that can effectively mitigate the risks estimated in this study.

The same is not true, however, for most other countries with high exposure, particularly low- and middle-income countries, where flood risks coincide with poverty and vulnerability. Vietnam, where 46% of the population is located in flood zones, is a leader among developing countries in its efforts to mitigate natural risks. Its extensive sea dike system stretches over 2600 kilometers, exceeding many other countries’ protective infrastructure^14^. Yet the system is built to safety standards that only protect against 1-in-30-year coastal flooding, and would be overwhelmed by more severe events^14^.

Geographic and urbanization patterns are driving the high flood exposure relative to countries’ population size. Considering exposure to different flood types highlights these factors (Fig. 3c–e). Fluvial flood risks dominate in areas where large population shares are concentrated in low-lying river basins, such as the Brahmaputra (Bangladesh), Euphrates and Tigris (Iraq), Irrawaddy (Myanmar), Indus (Pakistan), Mekong (Cambodia, Laos, Vietnam), and Nile (Egypt, South Sudan). Pluvial flooding drives risks in mountainous regions where natural drainage capacity is more limited and flash flood risks are heightened (e.g., Nepal, Andorra), or in climates with intense rainy seasons that exceed drainage and soil absorption capacity (e.g., Bangladesh, Guyana, Myanmar, Suriname). Coastal flooding dominates in countries with expansive coastal urbanization (e.g., Guyana, Vietnam) and islands countries (e.g., The Bahamas, Fiji).

### Flood exposure at subnational level

**Fig. 4:**
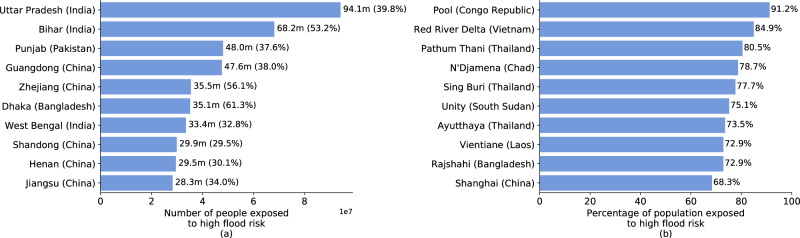
Absolute and relative population exposure at subnational level. **a** shows the ten subnational administrative areas with highest absolute number of people exposed (and as a percentage of the total population in parentheses). **b** shows the ten ADM-1 areas with highest relative population exposure.

A spatially disaggregated view of flood exposure estimates highlights that, within countries, risks are concentrated in specific areas, such as the coast or river basins. Several subnational regions stand out with large, exposed populations (Fig. 4a). In the Indian states of Bihar, Uttar Pradesh, West Bengal—all located along the Ganges River—a combined 196 million people live in high-risk flood zones, accounting for 33–53% of the states’ respective populations. In Pakistan, ~48 million of Punjab’s 120 million people live in high-risk flood zones, corresponding to 38% of the province’s total population. Located at the confluence of the Ganges and Brahmaputra Rivers, almost two-thirds of the population of Bangladesh’s Dhaka Division are directly flood-exposed. In China, exposed populations are largest in provinces along the coast and Yellow River Valley.

While these are all large subnational regions that often exceed the size of smaller countries, our results show that in smaller subnational areas, much larger population shares can be at risk (Fig. 4b). The world’s top 10 subnational areas in terms of relative exposure are all in Africa and Asia. Pool Department in the Republic of Congo is located along the Congo River, and we estimate that 91% of its population of 360,000 faces significant flood risk. The subnational areas with highest relative exposure in Africa are Chad’s capital region N’djamena, on the Chari River, and South Sudan’s Unity State, on the White Nile. In three Thai provinces, all located along the flood-prone Chao Phraya River, 70–80% of the population are at direct risk. With about 85% of their population living in flood zones, Vietnam’s Red River Delta provinces have some of the world’s highest exposure rates, and are the country’s main population and economic centers.

### Economic risk, poverty, and flood exposure

Using the World Bank’s global collection of harmonized household survey data, this study is able to highlight two seemingly contrasting findings: monetary flood exposure emphasizes risks in high-income countries; yet the interaction of flood exposure and poverty emphasizes risk in low-income countries. In short, by relying solely on monetary risk estimates, planners would bias their attention toward areas with high-value assets and large resources. But in so doing, they risk overlooking areas with high socioeconomic vulnerability, where flood risk mitigation measures are most urgently needed to protect lives and livelihoods.

**Fig. 5:**
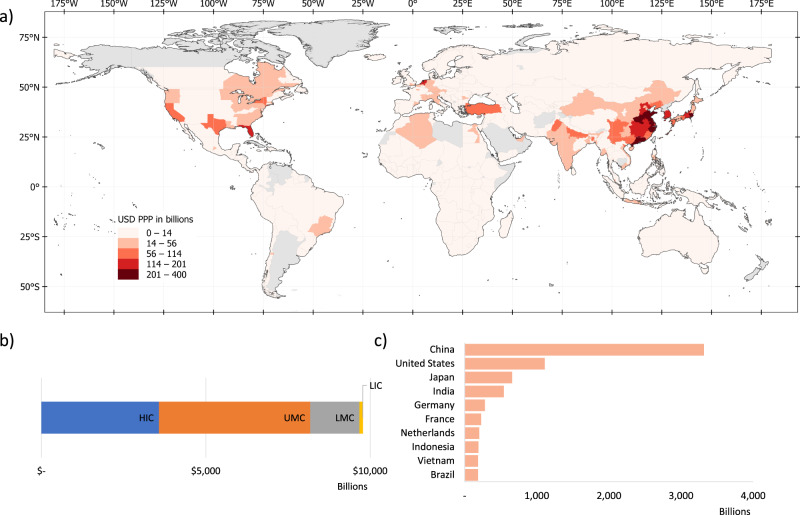
Economic activity at risk, estimated by exposure headcounts multiplied by subnational income per capita. **a** shows the economic value at risk at an ADM-1 level. **b** displays the total economic value at risk for each region. **c** lists the top 10 countries with the highest economic value at risk. HIC high-income countries, UMIC upper middle-income countries, LMIC lower-middle-income countries, LIC low-income countries.

By combining the headcount estimates with per capita income levels, we translate flood exposure headcounts into estimates of the economic activity value that is directly exposed to flood risk. This monetary risk estimate suggests that $9.8 trillion of economic activity is directly located in areas with significant flood risks (note that this refers to exposed, not lost, economic activity, and does not distinguish people’s place of residence and work). This is equivalent to about 12% of global gross domestic product (GDP) in 2020^30^. As Fig. 5 illustrates, monetary risk estimates highlight risks in higher-income countries, with the highest economic exposure in North America, Europe and East Asia, and Sub-Saharan Africa classified as having “low exposure” in monetary terms.

Of the $9.8 trillion of economic activity in flood risk areas, 84% is located in high- and upper middle-income countries (following the World Bank’s income classification). High-income countries account for 37% of exposed economic activity, but only 11% of the world’s flood-exposed population. In contrast, low- and lower-middle-income countries account for 52% of exposed people, but only 16% of exposed economic activity. Among countries with the largest economic value at risk, China leads, with $3.3 trillion exposed, followed by the USA ($1.1 trillion) and Japan ($0.7 trillion); no low-income country is among the top 10 countries in terms of economic value at risk. In interpreting these results, it is important to note that flood risk exposure does not account for existing flood protection measures. Such measures tend to be better developed in high-income countries, meaning that the fraction of exposed economic activity lost during a flood tends to be higher in low-income countries^1^.

Floods have been documented to cause more long-lasting and devastating effects in low-income communities. Here, lower-quality buildings and assets mean damages are higher^1^; inadequate planning and drainage infrastructure exacerbate hazards; the lack of widespread formal banking means people cannot draw on liquid savings or affordable credit to cope and recover; social systems lack the resources and reach they need to support affected populations; and insurance markets are less developed. To understand where flood risks pose the largest threat to development outcomes, a systematic assessment of poverty rates is essential.

**Fig. 6:**
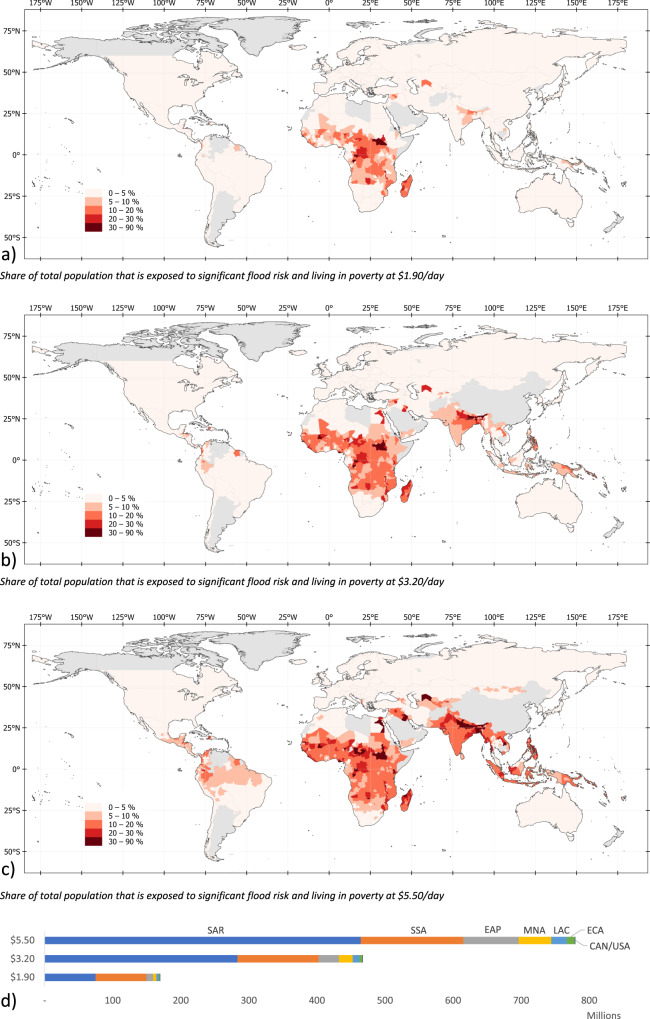
Poverty and flood exposure. **a**–**c** chart the share of total population exposed to significant flood risk and living with income below $1.90, $3.20, and $5.20 per day, respectively. **d** shows for each region the total number of people who are both exposed to significant flood risk and living in poverty. EAP East Asia and Pacific, ECA Europe and Central Asia, LAC Latin America and the Caribbean, MNA Middle East and North Africa, SAR South Asia region, SSA Sub-Saharan Africa.

Our estimates show that of the 1.81 billion flood-exposed people globally, at least 170 million are living in extreme poverty (i.e., on less than $1.90 per day). Of these, 88% are located in Sub-Saharan Africa and South Asia (Fig. 6). Flood exposure coincides with poverty most widely in Sub-Saharan Africa, where 74.7 million people are both flood-exposed and living in extreme poverty; in South Asia, the figure is also 75.0 million, driven by India (66 million).

The World Bank defines the $1.90 a day threshold as the most severe form of poverty, corresponding to a minimum subsistence level in low-income countries. However, floods are major livelihood shocks for all affected low-income households, even if they do not fall under the extreme $1.90 line. Hence, and given persistent poverty in middle-income countries, it is essential to consider less extreme poverty definitions. Indeed, when using less stringent poverty thresholds, the number of flood-exposed people in poverty increases significantly. We estimate that, globally, around 467 million people live in high-risk flood zones while living on less than $3.20 a day, increasing to 780 million if we consider incomes under $5.50 a day. This means that four out of every ten people exposed to flood risk globally are living in poverty (Table 1).

**Table 1:** Global flood exposure headcounts at different poverty thresholds for 2020.

	Poverty threshold (consumption per day)		
	$1.90	$3.20	$5.50
Number of people in poverty (millions)	741.8	1812.0	2986.6
Share of global population living in poverty (%)	9.4	23.0	38.0
Number of that are flood-exposed and live in poverty (millions)	170.0	467.4	779.7
Share of people that are flood-exposed and live in poverty (%)	22.9	25.8	26.1
Share of global population that is flood-exposed and lives in poverty (%)	2.2	5.9	9.9

The maps in Fig. 6 highlight that raising the poverty threshold shifts the geographic concentration of poverty and flood exposure from mainly Sub-Saharan Africa to include subnational regions in Egypt, the Middle East, South and East Asia, and Latin America. Increasing the poverty threshold from $1.90 to $5.50 doubles the number of people in Sub-Saharan Africa facing flood exposure and poverty from 75 million to 151 million. In SAR, the increase is sixfold, from 75 million to 464 million; in East Asia, it is eightfold, from 10 million to 81 million.

**Fig. 7:**
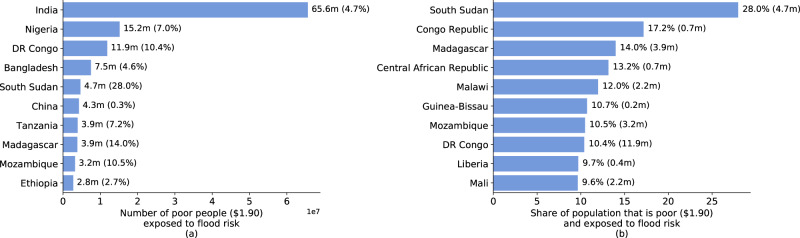
Hotspots of coinciding poverty and flood exposure. Top 10 countries in terms of the number (**a**) and share (**b**) of people who are exposed to significant flood risk while living in extreme poverty (at $1.90/day).

Among the top 10 countries where extreme poverty (at $1.90 threshold) and flood exposure coincide, seven are in Sub-Saharan Africa (Fig. 7a). With over 65 million, India has the highest number people exposed to flood risk and living in extreme poverty, though this represents only 16.8% of its total exposed population (390 million). As a share of the overall population, extreme poverty and flood exposure coincide most acutely in Sub-Saharan Africa (Fig. 7b); for these countries, 9–28% of the population faces significant flood risk while living in extreme poverty.

Overall, these results highlight that flood risks are substantial in many low-income countries. Our results also show that the risks are often concentrated in subnational regions within these countries—for example, there are provinces in South Sudan and Congo where over 50% of the population is both flood exposed and living in extreme poverty. Despite being typically overlooked by monetary measures of flood risk, these countries and regions face substantial vulnerabilities due to poverty and associated challenges surrounding social safety nets and infrastructure quality.

## Discussion

This study presents global estimates of the number of people exposed to high flood risks, while accounting for their respective poverty levels. To this end, we use the latest global high-resolution data on different flood hazard types (fluvial, pluvial, and coastal) and population density. This is combined with subnationally disaggregated poverty data, based on the World Bank’s global database of harmonized household consumption surveys. These data sources enable us to conduct a high-resolution global flood hazard assessment, processing over 38 billion data points, covering 7.9 billion people in 188 countries and 2084 subnational regions.

Our estimates show that about 1.81 billion people, or 23% of the world population, are directly exposed to inundation depths of over 0.15 meter during 1-in-100-year floods, which would pose significant risk to lives, especially of vulnerable population groups. This figure significantly exceeds previous estimates and highlights that past studies have offered only a partial picture: either because they focus on a single flood type (e.g., sea level rise), have limited geographic coverage, or lack high-resolution data, thus failing to capture the localized nature of flood risk.

The majority of flood-exposed people (1.24 billion) are located in South and East Asia, where China (395 million) and India (390 million) account for over one-third of global exposure. In several subnational areas of South and East Asia, more than two-thirds of the population is exposed to significant flood risks. Yet, the estimates also highlight that flood risks are a near universal threat, affecting people in all countries covered in this study. Of the 2084 subnational regions analyzed in this study, only 9 have less than 1% of their population exposed to flood risks.

However, flood exposure headcounts alone cannot offer a full picture. It is crucial we also consider the income levels of flood-exposed populations, as these can act as a proxy for people’s ability to mitigate, withstand, cope with, and recover from floods. For instance, while a large share of the Dutch population lives in flood risk areas, large-scale investments in flood protection infrastructure have enabled them to mitigate risks. Similarly, flood-exposed populations in Canada or Japan are more likely to have access to rapid government support systems in postdisaster situations compared to people in Malawi or Bangladesh. Floods in low-income countries are documented to have long-lasting and devastating impacts on livelihoods. Thus, action to strengthen disaster prevention and recovery capacity is most urgently needed in the hotspots where poverty and flood exposure coincide.

We show that flood risks are most prevalent in the developing world, as low and middle-income countries are home to 89% of the world’s flood exposed. We also estimate that globally, 170 million to 780 million people are exposed to flood risks while living in poverty (depending on the poverty definition used). By assessing the coincidence of flood risks and poverty, we highlight regions where flooding is likely to cause the most detrimental impacts on livelihoods and well-being. By this measure, countries in Sub-Saharan Africa face the greatest threat: we estimate that, of the 176 million flood-exposed people in this region, at least 75 million live in extreme poverty (i.e., on less than $1.90 a day). In addition, by using a less stringent $5.50 a day poverty definition, we highlight the risks to low-income communities in South Asia, where 464 million people face flood risks while living in poverty.

We conclude that flooding is a risk with global prevalence, and systematic risk mitigation measures are crucial to prevent the loss of lives and livelihoods and reversals of development progress. We show that, under current conditions, more people than previously known are exposed to flood risks. Climate change and risky urbanization patterns are expected to further aggravate these risks in coming years^3,5,31,32^. Yet, when prioritizing flood protection investments, focusing on the monetary exposure of assets and economic activity is bound to bias attention toward high-income countries and economic hubs. By accounting for the poverty levels of exposed populations, we show that low-income countries are disproportionately exposed to flood risks, while being more vulnerable to disastrous long-term impacts. To facilitate prioritization and comprehensive action, our findings highlight the scale and priority regions for flood risk mitigation measures to support resilient development.

## Methods

### Global flood hazard data

To obtain complete estimates of global population exposure to flood risk, this study considers the three most common flood types:

- Fluvial flooding, which occurs when intense precipitation or snow melt causes rivers to overflow.
- Pluvial flooding, which occurs when rainwater builds up beyond the absorptive capacity of soil.
- Coastal flooding, which is caused by storm surges and high tides in coastal areas.

Country-level pluvial and fluvial flood data are based on the 2019 version of the global Fathom flood hazard dataset^33,34^. The datasets provide gridded information on flood extents and flood depths at a 3 arcsecond (equivalent to 90 meters at the equator), simulating flood events with return periods of 5, 20, 50, 100, 250, and 500 years, and are available for all countries. The maps are based on the DEM MERIT elevation model that corrects for multiple errors, including absolute bias, stripe noise, speckle noise, and tree and building height biases^35^. We consider flooding with a 100-year return period; and use the undefended flood maps, which do not incorporate the effects of artificial flood protection structures. This is likely to result in overestimation of exposure in locations where flood protection systems defend against 100-year floods (or higher). Since no complete global inventory of flood defense structures exists, it is not possible to accurately assess the size of this overestimation. However, case studies and World Bank country risk assessments suggest that the vast majority of flood-exposed people in low- and lower-middle-income countries have no protection at all from the flood intensity considered in this study^1,14,36^. Many low-income countries lack even basic drainage systems to manage light flooding^1,4^. Similarly, it is likely that only high- and some higher middle-income countries offer such flood protection standards to a significant share of their populations—but even here, frequent flood disasters demonstrate that such coverage is far from complete.

We use a global coastal flood risk map with 3 arcsecond resolution, generated using the LISFLOOD-FP hydrological model^37,38^. As with the fluvial and pluvial flood maps, it relies on DEM MERIT as an input to the model^39^. Coastal flood simulations are forced by extreme sea level scenarios derived from reanalysis of waves (using the WAVEWATCH-III model^40^ and storm surges (using the DFLOW-FM model^41^, and further combined with tidal information^42^. Vousdoukas et al. provide further details on the coastal flood modeling^38^. As with the fluvial and pluvial floods, we consider 100-year coastal flood events.

### Population data

We estimate the location of people using the WorldPop Global High Resolution Population dataset (WPGP), produced by the University of Southampton, the World Bank, and other partners. It offers global coverage and is available yearly from 2000 to 2020. While WorldPop provides several datasets (including poverty, demographics, and urban change mapping), we use the population density map (WorldPop-PPP-2020). In a raster format, this dataset provides the number of inhabitants per cell, with a resolution of 3 arcseconds, thus specifying the distribution of population. This information is based on administrative or census-based population data, disaggregated to grid cells based on distribution and density of built-up area, which is derived from satellite imagery^43,44^.

The choice of population density map is important for the purpose of this study. Smith et al. provide a sensitivity analysis for flood exposure assessments using different population density maps, including WorldPop (3 arcsecond)^22^. They show that high-resolution population density maps perform best in capturing local exposure distribution, particularly the High-Resolution Settlement Layer (HRSL) with 1 arcsecond, or ~30 m resolution, produced jointly by Facebook, Columbia University and the World Bank^45^. While HRSL is only available for a limited number of countries, WorldPop is shown to perform better than alternatives with global coverage, such as LandScan data (30-arcsecond, ~900 m resolution)^46^.

### Return periods

Natural hazard data typically distinguish return periods to describe the probability with which a natural shock of a certain spatial distribution and intensity can be expected to occur. Based on historic data and the statistical frequency of a shock of a certain intensity, a return period describes how much time is expected to pass before a natural shock of the same intensity occurs again. For example, a flood event with a 25-year return period (or a 1-in-25-year flood) has a 1/25 or 0.04 annual probability of occurring. In other words, each year there is a 4% chance of such an event occurring, regardless of when the last such event took place. By extension, there is a 63.9% cumulative probability that a flood of at least this intensity will occur once within a 25-year period. But this also leaves the possibility for this event to not occur at all, or to occur several times. In comparison, a 1-in-100-year flood has a lower probability but higher intensity.

This study considers flood hazards with a 100-year return period. Such 1-in-100-year floods have, on average, a 1% probability of occurring in any given year, which translates to 10% in a decade, or 50% in a lifetime (68 years). These are significant probabilities that lie well within the planning horizons of governments. For instance, the US National Flood Insurance Program makes flood insurance compulsory for all buildings in 1-in-100-year (or riskier) flood zones. It should also be noted that these probabilities apply independently to a given river basin or microclimate. For the purpose of this study, we consider hundreds of thousands of such locations. This means that, globally, hundreds of 1-in-100-year flood events happen every year. As time passes, more climatic data become available, which will update the empirical probabilities associated with certain natural shocks.

### Administrative boundaries and poverty estimates

For the purpose of this study, national boundaries are further disaggregated into subnational units for which statistically representative poverty estimates are available from the World Bank’s GSAP. We use the GSAP’s latest update, produced in 2020. These subnational units are typically provinces or states (Admin-1), but can also include custom groupings of subnational regions determined by the sampling strategy of household surveys. Overall, this study covers 188 countries, disaggregated into 2260 subnational units. For each subnational unit, the GSAP offers several poverty and income estimates, which are derived from countries’ latest available household survey, in particular the Living Standards Measurement Surveys. By applying poverty rates at the subnational administrative level, we assume that hazard exposure is uniform across income groups within that given area. This may result in an underestimate of flood exposure if local sorting results in disproportionate exposure of low-income households. For instance, if flood risks are reflected in land prices, lower-income groups may be forced into riskier cheaper areas. Thus, this study’s estimates on the flood exposure of people in poverty should be interpreted as lower bound estimates. For this study, we use the standard World Bank definitions of poverty used to determine poverty headcounts in a given subnational administrative unit, based on the daily expenditure thresholds of $1.90, $3.20, and $5.50. All $-values in this study denote USD.

### Estimation of population exposed to flood risk

To estimate the number of people who are exposed to intense flood risk, this study follows four main steps:

In the first step we generate a combined global flood hazard map. For each country and subnational administrative unit, we create a single flood hazard layer by combining different flood types. The resulting flood map has a 3 arcsecond resolution, with each grid cell indicating estimated inundation depths in meters. For pixels where different flood types overlap—such as coastal areas near rivers that are exposed to both coastal and fluvial flooding—we retain the higher inundation depth estimate. We then resample the flood hazard map to ensure that pixels align with the WorldPop population density map.

Second, we define flood risk categories. While the flood hazard map offers inundation depths along a continuous scale, we aggregate the values into five risk categories, defined in line with an approximation of the risk to the lives and livelihoods of affected people: (1) “No risk”: areas that are estimated to remain unaffected during 100-year floods; (2) “Low risk”: inundation depths of up to 0.15 meters, no significant expected risk to life or economic activity; (3) “Moderate risk”: inundation depths of up to 0.5 meters, bearing disruptions to livelihoods and economic activity, as well as some risk to life for select locations and population sub-groups, especially among vulnerable groups such as children and disabled people; (4) “High risk”: inundation depths of up to 1.5 meters, a significant share of the affected population is expected to face risk to life, especially if flood waters have a current, and major disruptions to livelihoods; (5) “Very high risk”: inundation depths above 1.5 meters, most affected people could face substantial risk to life and severe and prolonged disruptions to livelihoods. In addition to these five categories, we denote flood risk to be “significant” when inundation depths are higher than 0.15 meters (i.e., combining “moderate” to “very high” categories)^47^. Through this process, we assign each grid cell to one of the five risk categories. We repeat this for the world’s landmass of about 149 million square kilometers, thus processing over 38 billion pixels.

In the third step we assign flood risk categories to population headcounts at the pixel level and aggregate results to the administrative unit level. For this purpose, we convert the flood hazard and population density maps into the same spatial resolution and assign each population cell a unique flood risk classification. We then aggregate cell level exposure headcounts to the administrative unit (e.g., province or district) level, thus yielding population headcounts for each flood risk category and (sub)national administrative unit. Hence, the estimated exposure headcounts of this study are available as gridded outputs with a resolution of 3 arcseconds, as well as aggregated to administrative units—including for each country and subnational unit, and regional and global estimates.

Lastly, we compute the number of people exposed to both flood risk and poverty. While poverty estimates are not available at the pixel level, the World Bank’s GSAP provides them at subnational level for most countries. We multiply these poverty shares with the exposure headcounts to obtain an estimate of the number of people in each administrative unit, who live below the poverty line and are exposed to flood risk (using the three poverty definitions cited above). Similarly, we multiply exposure headcount estimates with subnational GDP per capita figures to obtain estimates of flood-exposed GDP in monetary terms. For China, mean income levels are only available at national, not subnational, level. All computations were conducted using Python, and visualizations using QGIS.

### Sensitivity of results

The exposure headcount estimates in this study are aggregated along five inundation depth thresholds. People located in areas with higher inundation depths during a flood are assigned a higher risk class. The overall headcount estimates in this study are sensitive to the choice of these thresholds. There is evidence that flood depths of at least 0.15 meters can already cause significant disruptions to economic activity and livelihoods, thus this study uses this as the lower bound threshold for classifying flood exposure. However, the standards of socioeconomic resilience and flood defense infrastructure differ from place to place, as does the accuracy of data. We thus report figures on the sensitivity around these thresholds: We estimate 1.81 billion people to live in flood zones with over 0.15 meter inundation depth. This headcount is reduced to 1.06 billion when considering only severe fluvial, pluvial, and coastal flooding of over 0.5 meters. A full sensitivity analysis that disaggregates three flood types and varies the lower bound threshold between zero and 1 meter shows that estimates for the exposure to pluvial flooding are most sensitive to threshold changes; the sensitivity of coastal flood exposure does not affect overall headcount estimates. Sensitivity figures are provided in Supplementary Note 2.

### Reporting summary

Further information on research design is available in the Nature Research Reporting Summary linked to this article.

## Supplementary information

Supplementary Information File

Reporting Summary

## Data Availability

Global fluvial and pluvial flood hazard data (July 2020 version) are used with the permission of Fathom Global. The coastal flood maps were developed and made available by the Joint Research Centre of the European Commission. The population density map (WorldPop-2020) is publicly available for download^29^. For each of the countries analyzed, results are freely available from the World Bank’s Development Data Hub as raster files 3 arcseconds spatial resolution and as shapefiles with data aggregated to the ADM-1 (subnational), and ADM-0 (national) levels. Full country-level results are also provided in Supplementary Table 1.

## Code availability

The source code for this study is available from https://github.com/junrent/Global_floods (10.5281/zenodo.6330694).
