# Supplementary material for: Flood exposure and poverty in 188 countries
Source: Nat Commun. 2022 Jun 28;13:3527. doi: 10.1038/s41467-022-30727-4 (PMC9240081; doi:10.1038/s41467-022-30727-4)
Supplement: Supplementary file 1 — Supplementary Information File [file 41467_2022_30727_MOESM1_ESM.pdf]

Supplementary Information:
Flood exposure and poverty in 188 countries

Jun Rentschler[1,2*], Melda Salhab[1,3], Bramka Arga Jafino[4,5]

Supplementary Note 1. Stepwise computational process

The following steps are repeated for each subnational region where data is available.

Merge coastal, pluvial, fluvial flood hazard maps (Supplementary Figure 1.)

- Crop fluvial and pluvial flood rasters to subnational (admin-1) boundary
- Crop and then virtually warp the coastal flood raster, using a nearest neighbors resampling method, to perfectly match the resolution and extent of the fluvial and pluvial rasters
- Merge fluvial, pluvial, and coastal rasters using a maximum value method

Coastal

0	0	0	0.1
0	0	0	0.12
0	0	0	1.6
0	0	0.7	999

Pluvial

1.2	0	0	0
0.8	0	0	0
0.4	0	0	0
0.14	0	0	0

Fluvial

1.6	0.9	0	0
0.6	0.02	0	0
0	0	0	0
0	0	0	0

Combined Flood Hazard

1.6	0.9	0	0.1
0.8	0.02	0	0.12
0.4	0	0	1.6
0.14	0	0.7	999

Supplementary Figure 1. Merger of three flood layers

Categorize the flood raster (Supplementary Figure 2.)

- Using the pre-defined flood categories and a category for water bodies, categorize the value of each pixel

Combined Flood Hazard

1.6	0.9	0	0.1
0.8	0.02	0	0.12
0.4	0	0	1.6
0.14	0	0.7	999

Categorized

4	3	0	1
3	1	0	1
2	0	0	4
1	0	3	5

Supplementary Figure 2. Flood risk categorization

Convert to Boolean integer raster (Supplementary Figure 3.)

- Convert the country raster into six different rasters, one for each flood bin
- Convert each raster for each flood bin to a Boolean (true / false) integer array, where 1 represents the presence of flood risk level in the specific pixel, and 0 represents no presence of flood risk for that pixel

[1] The World Bank, Washington DC, USA
[2] Payne Institute for Public Policy, Denver, USA
[3] Center for Advanced Spatial Analysis, University College London, London, UK
[4] Deltares, Delft, The Netherlands
[5] Faculty of Technology, Policy, and Management, Delft University of Technology, Delft, The Netherlands

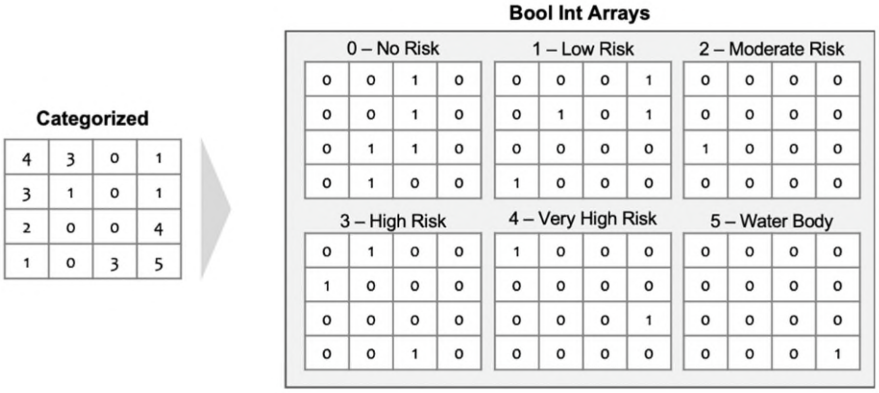

Supplementary Figure 3. Separating flood categories

Multiply each flood array by the population array (Supplementary Figure 4.)

- Crop and virtually warp the population raster to perfectly match the flood raster
- For each flood risk array, multiply the array by the population
- Each flood array pixel is multiplied by the corresponding population array pixel
- The 1/0 structure allows the resulting array to capture population data for the specific flood risk level

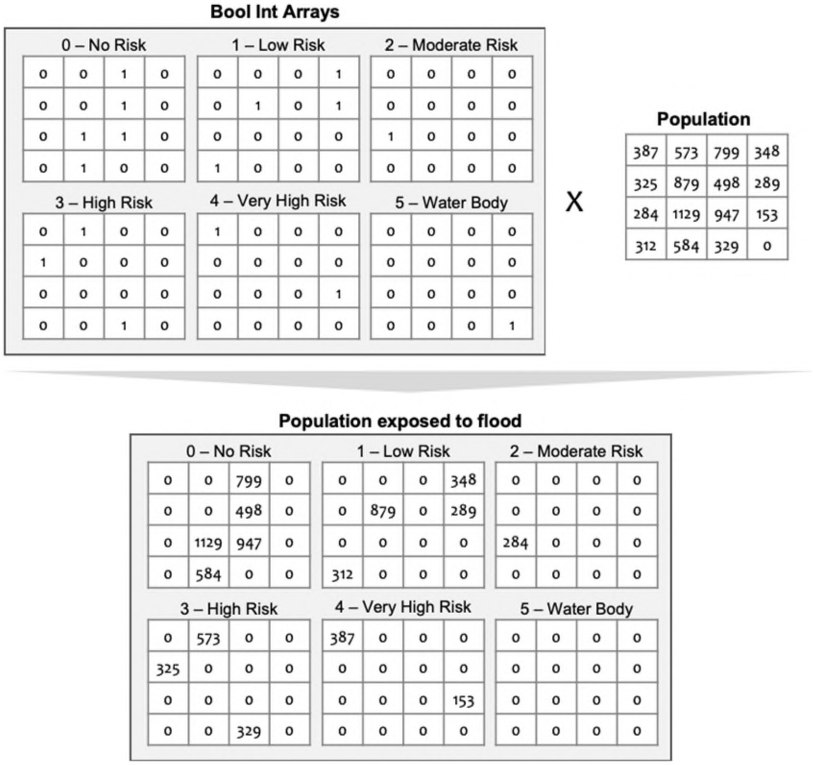

Supplementary Figure 4. Overlay of flood risk and population layers

Calculate sums

- For each flood risk array, calculate the total number of people exposed and add the results to the World Bank global administrative map shapefile

Supplementary Note 2. Sensitivity of results to threshold choice

Supplementary Figure 5. shows the number of people who are exposed to pluvial, fluvial, or coastal flooding of at least a certain inundation depth during a 1-in-100 year event. Headcount estimates for the exposure to pluvial flooding are most sensitive to threshold changes. The sensitivity of coastal flood exposure does not significantly affect the overall exposure headcount estimates (from 165 million people when threshold is set at 15 cm to 132 million people with a threshold of 100 cm). Note that locations are often exposed to more than one flood type, hence summing exposure headcounts of individual flood types would result in double counting.

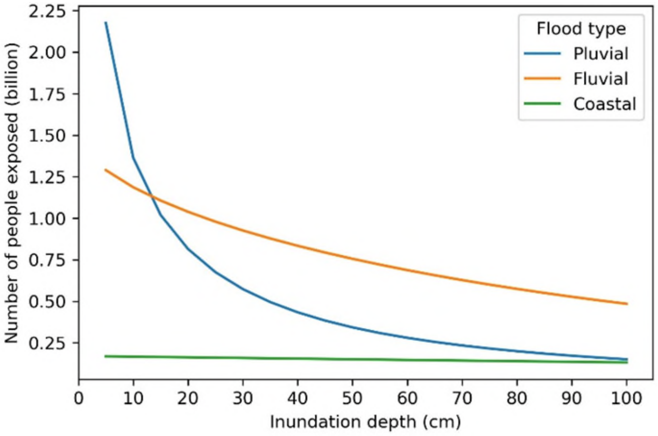

Supplementary Figure 5. Sensitivity analysis of global flood exposure to individual flood types

Supplementary Note 3. Country level summary results

Supplementary Table 1. Exposure headcount estimates at the country level

		**Total population**	**Population exposed to high flood risk**		**Population exposed to high flood risk and poor ($1.9 per day)**		**Population exposed to high flood risk and poor ($3.2 per day)**		**Population exposed to high flood risk and poor ($5.5 per day)**	
		('000)	Total *('000)*	% of population	Total *('000)*	% of population	Total *('000)*	% of population	Total *('000)*	% of population
1	Afghanistan	30,296	4,501	14.9	0	0.0	0	0.0	0	0.0
2	Albania	2,797	771	27.6	8	0.3	50	1.8	240	8.6
3	Algeria	44,084	4,875	11.1	16	0.0	110	0.2	1,021	2.3
4	Andorra	113	28	25.0	0	0.0	0	0.0	0	0.0
5	Angola	36,246	3,732	10.3	1,797	5.0	2,614	7.2	3,280	9.1
6	Antigua and Barbuda	93	9	9.9	0	0.0	0	0.0	0	0.0
7	Argentina	44,827	7,215	16.1	0	0.0	0	0.0	0	0.0
8	Armenia	2,773	406	14.6	5	0.2	37	1.3	173	6.3
9	Australia	20,804	1,654	7.9	8	0.0	13	0.1	13	0.1
10	Austria	8,761	2,437	27.8	8	0.1	10	0.1	16	0.2
11	Azerbaijan	8,646	1,520	17.6	0	0.0	0	0.0	0	0.0
12	Bahamas	385	81	21.1	0	0.0	0	0.0	0	0.0

13	Bangladesh	164,109	94,424	57.5	7,504	4.6	38,612	23.5	73,349	44.7
14	Barbados	284	20	6.9	0	0.0	0	0.0	0	0.0
15	Belarus	9,157	882	9.6	0	0.0	0	0.0	4	0.0
16	Belgium	11,580	2,070	17.9	2	0.0	3	0.0	5	0.0
17	Belize	414	88	21.4	10	2.4	21	5.1	40	9.8
18	Benin	12,990	1,950	15.0	899	6.9	1,433	11.0	1,772	13.6
19	Bhutan	813	63	7.7	0	0.1	5	0.6	20	2.4
20	Bolivia	11,832	1,875	15.8	66	0.6	165	1.4	381	3.2
21	Bosnia and Herzegovina	3,324	635	19.1	0	0.0	2	0.0	13	0.4
22	Botswana	2,401	294	12.2	39	1.6	104	4.4	176	7.3
23	Brazil	216,999	25,408	11.7	1,256	0.6	2,579	1.2	5,491	2.5
24	Brunei Darussalam	453	115	25.4	0	0.0	0	0.0	0	0.0
25	Bulgaria	6,892	1,184	17.2	14	0.2	34	0.5	85	1.2
26	Burkina Faso	22,781	3,289	14.4	1,148	5.0	2,339	10.3	2,971	13.0
27	Burundi	11,346	1,252	11.0	974	8.6	1,153	10.2	1,224	10.8
28	Cabo Verde	508	27	5.3	1	0.1	3	0.6	10	1.9
29	Cambodia	19,523	7,431	38.1	0	0.0	0	0.0	0	0.0
30	Cameroon	28,043	5,348	19.1	1,468	5.2	2,592	9.2	3,803	13.6
31	Canada	36,782	3,450	9.4	7	0.0	17	0.0	27	0.1
32	Cayman Islands	73	12	16.3	0	0.0	0	0.0	0	0.0
33	Central African Republic	5,347	1,036	19.4	704	13.2	867	16.2	967	18.1
34	Chad	16,589	4,547	27.4	1,591	9.6	2,804	16.9	3,797	22.9
35	Chile	20,672	3,484	16.9	10	0.0	22	0.1	116	0.6
36	China	1,437,455	394,826	27.5	4,289	0.3	0	0.0	0	0.0
37	Colombia	63,257	14,507	22.9	577	0.9	1,578	2.5	3,981	6.3
38	Comoros	846	74	8.7	11	1.4	28	3.3	49	5.7
39	Congo Republic	3,997	1,170	29.3	686	17.2	907	22.7	1,057	26.4
40	Costa Rica	4,817	948	19.7	16	0.3	41	0.8	121	2.5
41	Cote d'Ivoire	26,537	3,484	13.1	866	3.3	1,894	7.1	2,808	10.6
42	Croatia	4,061	1,094	26.9	6	0.1	11	0.3	35	0.9
43	Cuba	11,114	2,026	18.2	0	0.0	0	0.0	0	0.0
44	Cyprus	1,286	145	11.3	0	0.0	0	0.0	0	0.0
45	Czech Republic	10,816	1,500	13.9	1	0.0	2	0.0	5	0.1
46	DR Congo	114,168	15,797	13.8	11,869	10.4	14,344	12.6	15,420	13.5
47	Denmark	5,888	541	9.2	0	0.0	0	0.0	1	0.0
48	Djibouti	1,153	73	6.3	18	1.6	34	3.0	54	4.7
49	Dominica	69	6	9.0	0	0.0	0	0.0	0	0.0

50	Dominican Republic	12,045	1,784	14.8	8	0.1	50	0.4	258	2.1
51	Ecuador	17,617	3,726	21.1	111	0.6	319	1.8	857	4.9
52	Egypt	96,019	38,871	40.5	1,628	1.7	12,331	12.8	29,363	30.6
53	El Salvador	6,739	1,132	16.8	17	0.3	94	1.4	309	4.6
54	Equatorial Guinea	1,295	211	16.3	0	0.0	0	0.0	0	0.0
55	Eritrea	4,163	391	9.4	0	0.0	0	0.0	0	0.0
56	Estonia	1,391	99	7.1	0	0.0	0	0.0	1	0.1
57	Eswatini	1,096	99	9.0	28	2.6	51	4.7	71	6.4
58	Ethiopia	103,809	11,586	11.2	2,752	2.7	6,955	6.7	10,209	9.8
59	Faeroe Islands	51	3	6.3	0	0.0	0	0.0	0	0.0
60	Fiji	699	162	23.1	0	0.1	6	0.8	34	4.8
61	Finland	5,606	612	10.9	0	0.0	1	0.0	1	0.0
62	France	67,353	11,632	17.3	0	0.0	2	0.0	5	0.0
63	Gabon	2,972	579	19.5	17	0.6	62	2.1	186	6.3
64	Gambia	2,395	370	15.4	40	1.6	146	6.1	275	11.5
65	Georgia	3,728	695	18.6	29	0.8	104	2.8	281	7.5
66	Germany	78,292	13,670	17.5	12	0.0	33	0.0	68	0.1
67	Ghana	32,954	4,716	14.3	583	1.8	1,364	4.1	2,564	7.8
68	Greece	10,221	1,314	12.9	11	0.1	20	0.2	57	0.6
69	Grenada	99	9	8.8	0	0.0	0	0.0	0	0.0
70	Guam	164	15	9.5	0	0.0	0	0.0	0	0.0
71	Guatemala	18,257	2,421	13.3	170	0.9	502	2.7	1,068	5.8
72	Guinea	12,167	2,306	19.0	491	4.0	1,259	10.3	1,973	16.2
73	Guinea-Bissau	1,763	301	17.1	189	10.7	248	14.1	278	15.8
74	Guyana	728	276	37.9	13	1.8	32	4.4	71	9.7
75	Haiti	14,625	2,553	17.5	614	4.2	1,268	8.7	2,002	13.7
76	Honduras	9,449	1,801	19.1	261	2.8	496	5.3	861	9.1
77	Hong Kong	6,032	720	11.9	0	0.0	0	0.0	0	0.0
78	Hungary	9,344	1,489	15.9	7	0.1	17	0.2	41	0.4
79	Iceland	360	53	14.9	0	0.0	0	0.0	0	0.0
80	India	1,405,691	389,816	27.7	65,646	4.7	222,969	15.9	334,064	23.8
81	Indonesia	280,224	75,696	27.0	2,630	0.9	15,964	5.7	39,962	14.3
82	Iran	80,139	11,738	14.6	34	0.0	313	0.4	1,653	2.1
83	Iraq	44,427	16,350	36.8	279	0.6	2,385	5.4	8,707	19.6
84	Ireland	5,482	738	13.5	0	0.0	3	0.1	4	0.1
85	Israel	8,557	639	7.5	1	0.0	3	0.0	13	0.2
86	Italy	61,665	8,504	13.8	92	0.1	120	0.2	192	0.3
87	Jamaica	2,721	395	14.5	7	0.3	37	1.4	118	4.3
88	Japan	125,792	36,060	28.7	270	0.2	350	0.3	455	0.4
89	Jordan	7,138	455	6.4	1	0.0	13	0.2	105	1.5

90	Kazakhstan	18,074	3,144	17.4	1	0.0	9	0.1	179	1.0
91	Kenya	55,137	6,566	11.9	2,369	4.3	4,339	7.9	5,714	10.4
92	Kuwait	3,522	301	8.5	0	0.0	0	0.0	0	0.0
93	Kyrgyz Republic	5,659	907	16.0	4	0.1	85	1.5	461	8.1
94	Laos	7,527	2,985	39.7	218	2.9	927	12.3	1,994	26.5
95	Latvia	2,456	554	22.6	4	0.2	8	0.3	17	0.7
96	Lebanon	9,337	872	9.3	0	0.0	1	0.0	16	0.2
97	Lesotho	1,885	151	8.0	41	2.2	75	4.0	111	5.9
98	Liberia	4,543	1,197	26.4	441	9.7	833	18.3	1,090	24.0
99	Libya	6,921	701	10.1	0	0.0	0	0.0	0	0.0
100	Lithuania	2,748	333	12.1	3	0.1	5	0.2	12	0.4
101	Luxembourg	590	69	11.7	0	0.0	0	0.0	1	0.1
102	Macedonia	2,097	397	18.9	16	0.7	31	1.5	72	3.4
103	Madagascar	27,625	5,379	19.5	3,862	14.0	4,703	17.0	5,168	18.7
104	Malawi	18,470	3,057	16.5	2,212	12.0	2,776	15.0	2,976	16.1
105	Malaysia	34,193	8,194	24.0	1	0.0	11	0.0	160	0.5
106	Mali	23,243	5,129	22.1	2,243	9.6	3,915	16.8	4,816	20.7
107	Malta	424	22	5.2	0	0.0	0	0.0	0	0.0
108	Mauritania	4,448	579	13.0	46	1.0	171	3.9	380	8.5
109	Mauritius	1,277	169	13.2	0	0.0	2	0.2	18	1.4
110	Mexico	140,328	24,089	17.2	408	0.3	1,533	1.1	5,434	3.9
111	Micronesia	59	2	3.6	0	0.4	1	1.3	1	2.4
112	Moldova	3,673	496	13.5	0	0.0	4	0.1	60	1.6
113	Monaco	0	0	0.0	0	0.0	0	0.0	0	0.0
114	Mongolia	3,159	458	14.5	2	0.1	23	0.7	120	3.8
115	Montenegro	603	110	18.3	1	0.1	6	1.0	15	2.4
116	Morocco	35,638	3,924	11.0	27	0.1	217	0.6	992	2.8
117	Mozambique	30,851	5,002	16.2	3,233	10.5	4,175	13.5	4,650	15.1
118	Myanmar	47,823	19,104	39.9	193	0.4	2,159	4.5	9,227	19.3
119	Namibia	2,438	423	17.3	68	2.8	147	6.0	241	9.9
120	Nepal	40,735	11,993	29.4	686	1.7	4,053	9.9	8,492	20.8
121	Netherlands	17,196	10,100	58.7	16	0.1	26	0.2	37	0.2
122	New Caledonia	270	46	17.0	0	0.0	0	0.0	0	0.0
123	New Zealand	4,393	623	14.2	0	0.0	0	0.0	0	0.0
124	Nicaragua	6,881	1,147	16.7	31	0.4	134	1.9	373	5.4
125	Niger	23,865	3,350	14.0	1,393	5.8	2,496	10.5	3,098	13.0
126	Nigeria	216,151	38,994	18.0	15,204	7.0	26,638	12.3	34,173	15.8
127	North Korea	23,224	5,735	24.7	0	0.0	0	0.0	0	0.0
128	Norway	5,223	456	8.7	1	0.0	1	0.0	2	0.0
129	Oman	3,587	184	5.1	0	0.0	0	0.0	0	0.0

130	Pakistan	230,904	71,786	31.1	1,161	0.5	17,304	7.5	46,890	20.3
131	Palau	25	2	6.1	0	0.0	0	0.0	0	0.0
132	Panama	4,058	625	15.4	10	0.2	31	0.8	82	2.0
133	Papua New Guinea	9,145	1,374	15.0	369	4.0	721	7.9	1,084	11.9
134	Paraguay	7,485	983	13.1	15	0.2	66	0.9	176	2.4
135	Peru	35,696	3,396	9.5	157	0.4	462	1.3	1,080	3.0
136	Philippines	105,153	30,483	29.0	1,305	1.2	7,367	7.0	17,115	16.3
137	Poland	39,207	5,280	13.5	17	0.0	27	0.1	59	0.1
138	Portugal	10,118	440	4.4	2	0.0	3	0.0	7	0.1
139	Puerto Rico	3,659	429	11.7	0	0.0	0	0.0	0	0.0
140	Romania	33,822	6,548	19.4	149	0.4	312	0.9	656	1.9
141	Russia	142,717	18,394	12.9	4	0.0	62	0.0	692	0.5
142	Rwanda	13,488	1,284	9.5	679	5.0	1,001	7.4	1,164	8.6
143	San Marino	32	2	6.5	0	0.0	0	0.0	0	0.0
144	Sao Tome and Principe	208	26	12.4	9	4.5	17	8.2	22	10.8
145	Saudi Arabia	36,811	2,437	6.6	0	0.0	0	0.0	0	0.0
146	Senegal	16,174	2,376	14.7	790	4.9	1,538	9.5	2,091	12.9
147	Serbia	6,977	1,404	20.1	1	0.0	10	0.2	87	1.3
148	Sierra Leone	6,782	1,296	19.1	626	9.2	1,055	15.6	1,233	18.2
149	Singapore	4,439	577	13.0	0	0.0	0	0.0	0	0.0
150	Slovakia	5,440	1,401	25.7	17	0.3	21	0.4	36	0.7
151	Slovenia	2,180	472	21.7	0	0.0	0	0.0	0	0.0
152	Solomon Islands	585	117	20.1	29	4.9	68	11.6	99	16.9
153	Somalia	11,002	1,831	16.6	0	0.0	0	0.0	0	0.0
154	South Africa	59,943	3,663	6.1	682	1.1	1,364	2.3	2,086	3.5
155	South Korea	50,972	10,351	20.3	26	0.1	51	0.1	103	0.2
156	South Sudan	16,743	5,437	32.5	4,686	28.0	5,215	31.1	5,399	32.2
157	Spain	51,999	5,859	11.3	38	0.1	61	0.1	120	0.2
158	Sri Lanka	21,387	4,140	19.4	30	0.1	354	1.7	1,502	7.0
159	St. Lucia	169	17	10.3	1	0.5	2	1.0	3	2.0
160	Sudan	42,574	8,778	20.6	1,006	2.4	3,789	8.9	6,875	16.1
161	Suriname	619	233	37.7	45	7.3	70	11.3	108	17.5
162	Sweden	10,064	978	9.7	2	0.0	2	0.0	5	0.0
163	Switzerland	8,693	1,660	19.1	1	0.0	0	0.0	1	0.0
164	Syria	26,550	3,548	13.4	1,603	6.0	2,730	10.3	3,294	12.4
165	Tajikistan	9,381	1,671	17.8	37	0.4	199	2.1	665	7.1
166	Tanzania	54,708	7,982	14.6	3,937	7.2	6,149	11.2	7,339	13.4
167	Thailand	74,946	25,431	33.9	5	0.0	109	0.1	1,765	2.4
168	Timor-Leste	1,322	317	24.0	62	4.7	198	15.0	286	21.6
169	Togo	8,631	1,349	15.6	621	7.2	978	11.3	1,211	14.0

170	Tonga	100	11	11.4	0	0.1	1	0.8	3	2.9
171	Trinidad and Tobago	1,377	291	21.1	1	0.1	3	0.2	18	1.3
172	Tunisia	11,707	1,334	11.4	6	0.0	62	0.5	319	2.7
173	Turkey	79,087	11,269	14.2	5	0.0	154	0.2	961	1.2
174	Turkmenistan	9,850	2,419	24.6	25	0.3	255	2.6	804	8.2
175	Turks and Caicos Islands	43	4	9.1	0	0.0	0	0.0	0	0.0
176	Uganda	42,049	5,438	12.9	2,287	5.4	3,834	9.1	4,788	11.4
177	Ukraine	43,674	5,407	12.4	1	0.0	19	0.0	175	0.4
178	United Arab Emirates	8,967	638	7.1	0	0.0	0	0.0	0	0.0
179	United Kingdom	66,928	7,390	11.0	18	0.0	30	0.0	41	0.1
180	United States	340,766	42,590	12.5	455	0.1	570	0.2	783	0.2
181	Uruguay	3,357	226	6.7	0	0.0	1	0.0	7	0.2
182	Uzbekistan	33,668	6,715	19.9	862	2.6	2,876	8.5	5,243	15.6
183	Vanuatu	272	30	11.1	4	1.5	12	4.5	22	8.2
184	Venezuela	31,718	5,843	18.4	0	0.0	0	0.0	0	0.0
185	Vietnam	98,839	45,504	46.0	465	0.5	2,133	2.2	8,843	8.9
186	Yemen	30,183	2,388	7.9	1,106	3.7	1,812	6.0	2,193	7.3
187	Zambia	18,684	2,611	14.0	1,621	8.7	2,024	10.8	2,327	12.5
188	Zimbabwe	14,360	1,426	9.9	468	3.3	869	6.1	1,157	8.1
